# Managing your eye unit's supplies

**Published:** 2011-12

**Authors:** RD Thulsiraj

**Affiliations:** Executive Director, LAICO, Aravind Eye Care System; President, VISION 2020: The Right to Sight: India, Lions Aravind Institute of Community Ophthalmology, Aravind Eye Care System, Annanagar, Madurai 625 020, Tamil Nadu, India.

**Figure:**
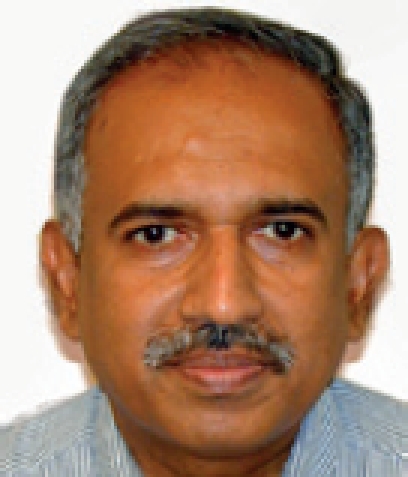
RD Thulsiraj

**Key learning points**

- Monitor your stock usage by keeping accurate records - this allows you to predict future usage and plan orders.
- The number of different items you stock can be limited through standardisation. It is desirable to do so as it may reduce cost and improve quality.
- Ensure uninterrupted services: keep enough stock to last until the next order arrives.
- Don't buy more stock than you will use before it expires.
- It is generally cheaper to make bigger orders, less frequently.
- Even if you make frequent, small orders, take advantage of bulk discounts by negotiating a price based on your projected annual usage. The actual orders can be delivered monthly or as required.
- Stock equals money. Money you spend on stock is money you can't spend on anything else, so buy as little as you can without risking an interruption to services.
- Invest in your supplies management systems - this will help you to perform better.

In order to deliver eye care, many resources have to be in place at the right time: your patients, your staff, your facilities, your equipment, and your supplies. In this article, we focus on how you can manage your supplies to ensure that your eye service runs smoothly. These supplies, or consumables, include every little thing needed in the course of your daily work: IOLs, medicines, gloves, forms used for patient care, housekeeping supplies, and equipment spares. If any of these items become unavailable, your eye centre will be unable to provide the same high quality of services, and you may even have to turn patients away.

The aim of good supplies management is to ensure uninterrupted services. If you want to effectively manage consumables in your eye unit, you will have to monitor the number of each item available (the ‘stock level’). To do this, you must keep an inventory.

## Keeping an inventory

An inventory is a document that lists all the items in stock that are required for the eye unit to function. It is the place where you record the quantity of each item in stock on a daily or weekly basis, depending on your needs. An inventory can be kept on paper or computer, or it can be kept using a combination of both. These days, several software systems are available for inventory management. They can also be integrated into computerised patient care or patient record systems.

**Figure:**
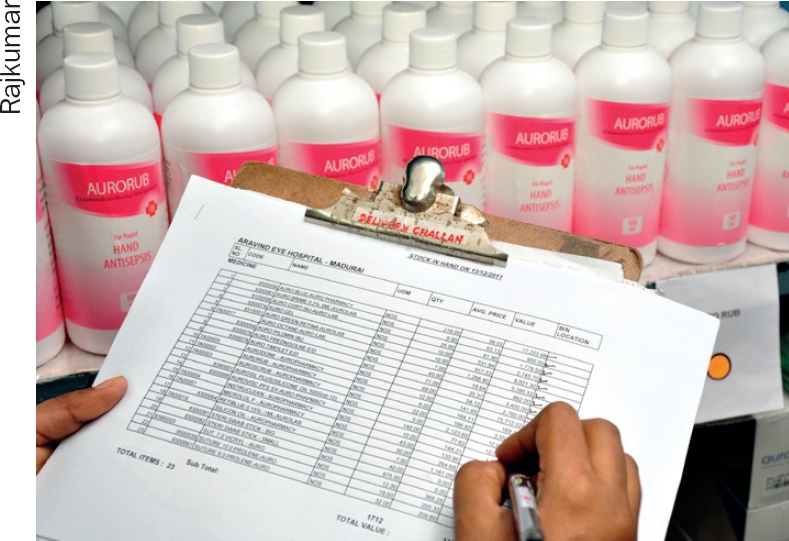
Physical verification of stock on hand

At any one time, the inventory must show the quantities of each item in stock, the purchase price of each item, and the shelf life of any consumables or medicines.

A good inventory system should be in place in every eye unit. To ensure the inventory is used properly, make it as easy and convenient as possible to work with. For example, the person in charge of managing the stores can keep a note of the stock levels of each product on an individual stock (or bin) card for each item, placed near the item in question. This can be used to update a general inventory register of all items, whether in a book or on computer (see the case study on page 35).

When items enter or leave the store, the stock card and inventory register must be amended accordingly. Ideally, staff should complete a form before taking any items, and hand a carbon copy of the form in to the stores manager.

Keeping an accurate inventory allows you to monitor how many of each item your clinic uses every week, or every month. This is very important to know, as it helps you to work out:

- What supplies to keep
- How much to keep
- How often to order new supplies
- How much to order each time.

**Figure:**
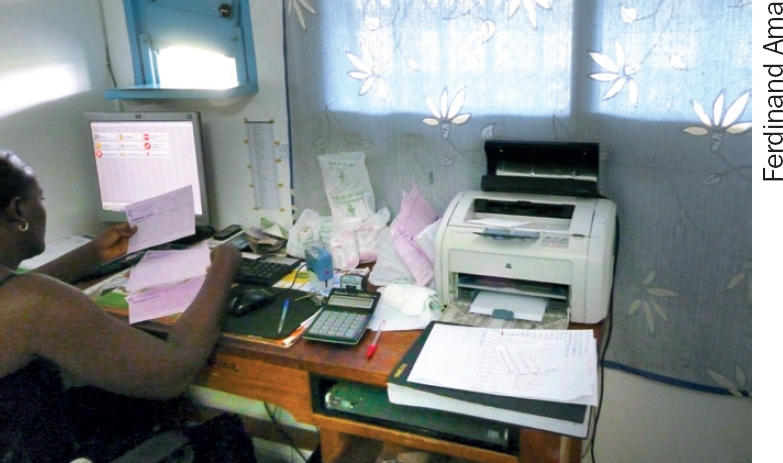
Entering stock levels on computer. IVORY COAST

**Stock cards**

**Figure 1:**
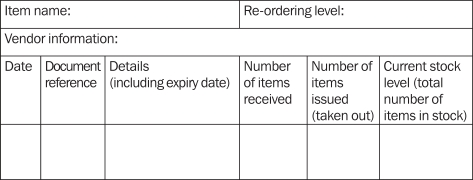
An example of a stock card

‘Re-ordering level’ refers to the minimum items you can safely have in stock before you order more (page 34). ‘Date’ refers to the date of the document related to the items leaving or entering the store, if available, or simply the date items entered or left the store. ‘Document reference’ is for the number, or any other identifying reference, on the supplier's invoice or on the internal authorisation for stock issued, as applicable. ‘Details’ is for writing a brief narrative, such as “Received from (supplier's name)” or “Issued to…”. Ideally, include the expiry date of any received stock in the ‘Details’ section.

**Figure:**
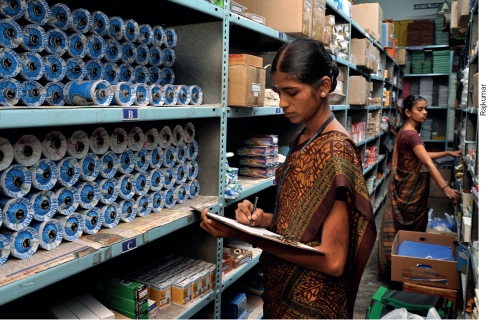
Shelves and racks can help organise your inventory. INDIA

## What supplies to keep

Your clinical protocols and administrative procedures determine to a large extent how many different items you need. For example, if your eye unit handles a wide range of conditions and has complex administrative procedures, you will need a larger number of items.

Also, where there is more than one ophthalmologist and each surgeon wants to use a different brand of IOL, this will increase the types and range of powers you stock. Stocking a large number of items can be more expensive and will make supplies management more complex, which has cost implications.

The number of items you stock can be limited through standardisation, where everyone agrees to use a limited but appropriate range of equipment, instruments, and consumables.

For standardisation to work, it is critical to involve the ophthalmologists in your eye unit. Everyone must agree which surgical supplies, equipment, instruments, and medications to use, including those used in outpatient services or outreach. The IAPB Standard List can help.

Your inventory will show which products are used less often and may be reconsidered. Other good questions to ask include:

- Which products are approved by the national government body concerned?
- Which products are easily available in our country?
- Which products meet our quality standards?

## How much to keep

Working out how much to keep is somewhat of a balancing act.

Too little can result in items being out of stock, leading to frequent disruption of work and emergency purchases, often at high costs. At the same time, playing it too safe, and stocking large amounts of supplies, means that you will need a larger storage facility, that you may risk higher wastage if the items expire, and that more of your eye unit's available cash will have been invested in stock, leaving you with less cash for other essential expenses.

The **minimum** number of each item you can safely stock is driven by two things: the number of patients you see, and how long it takes for new supplies to arrive from the day they are ordered.

Changes in the number of patients have a direct impact on how quickly you will use up your supplies. For instance, if more patients tend to come at a certain time of year, or if a training course is to take place, a larger number of items will be required.

When items are not locally available, it makes sense to stock them in larger quantities, particularly if the effort involved in ordering or purchasing is high and the delivery time is unpredictable or slow.

**‘Working out how much to keep is a balancing act’**

However, it is important to recognise that most medical supplies come with a specific shelf life and must be consumed before their respective expiry dates.

The **maximum** number of items you should sensibly stock is determined by:

- How much space you have to store supplies
- How quickly supplies will exceed their expiry date
- How much of your eye unit's cash you are willing to spend on stock.

## How much to order — and how often

There are costs and effort involved every time you order supplies. As we have seen, it also costs to hold stock by way of the money invested in the stock and the storage facilities required. The quantity of items ordered may also affect the price.

How often you order will determine how much you will order. For example, if the decision is to order an item every month, then the order quantity for that item would be equal to how much you use per month, on average.

It therefore makes sense to choose an optimum pattern of ordering that will balance all these costs.

There are two different approaches:

1. **Monthly replacement.** To start with, keep enough of each item in stock to last for two or three months. Then place orders every month for whatever has been used since the last ordering date. The work load of monthly ordering can be distributed over several days: one week it could be printed materials, another week it could be medicines or sutures, another week it could be maintenance and housekeeping materials. This way, not only is the ordering work load spread out, but the work related to receiving, verifying, and accounting will also be spread out. This would work well for most secondary level, or district, eye units.
  **TOP TIPS: Supplies management**
  - **Reduce the effort involved in making frequent orders.** Once you have fixed a price with your supplier, decide how much you want to keep in stock and negotiate with the supplier for weekly or monthly replacement. It will eliminate the whole ordering process and shift the responsibility to the supplier.
  - **Employ a competent stores manager.** This person should have basic training in supplies management and should ideally be computer literate. The store manager can make a valuable contribution to the efficient and cost-effective running of the eye unit.
  - **Go digital.** If you can afford it, a computerised supplies management system can work together with the manual stock entry system. This will show your stock balance, consumption, suppliers, prices, discounts, expiry dates, re-order levels, and re-order quantities.
  - **Monitor other stocks.** Check **theatre stocks** before surgery begins and ensure that all needed items are delivered to the operating room to avoid interruptions during surgery. There may be some medicines held in the **pharmacy** or in an **outreach box.** Check these regularly to ensure that they are in date.
  - **Arrange with the supplier to replace drugs with an early expiry date** with ones that have a later expiry date. This will reduce wastage.
  Contributed by RD Thulsiraj, Demissie Tadesse, and Pierre Ngounou.
2. **Set re-ordering levels for individual items.** Calculate re-ordering levels (or minimum stock levels) for individual items. When stocks are reduced to that level or quantity, the item is re-ordered (see the worked example below). This approach relies on having good supplies management systems and can work well in larger hospitals. Smaller hospitals could also use this system for items that are not locally available and which may take longer than a month to arrive.

## Bulk buying

The quantity of items bought can also affect the price: the larger the order, the cheaper the price of each item in the order (a ‘bulk discount’). Even if you decide to order frequently, and hence have smaller orders, you can still take advantage of bulk discounts by negotiating with the supplier to fix the price based on your annual consumption estimate - this is known as a framework agreement. The actual orders can be delivered monthly or as required.

The quantity ordered also affects the price of printed items that are customised to your hospital or eye centre, like the forms and letterheads which bear the hospital's name. Printing them in small quantities is very expensive and may not be an attractive order for the supplier.

**Figure:**
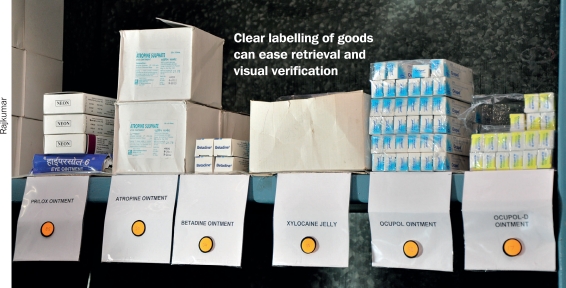


## Arranging your stock

When new supplies of a particular product arrive, move those with the shortest shelf life to the front of the shelf and put those with the longest shelf life at the back. This is the ‘first in, first out’ approach. Some medicines have to be kept in cold storage. Check labels carefully and make the appropriate cold storage arrangements.

**Figure:**
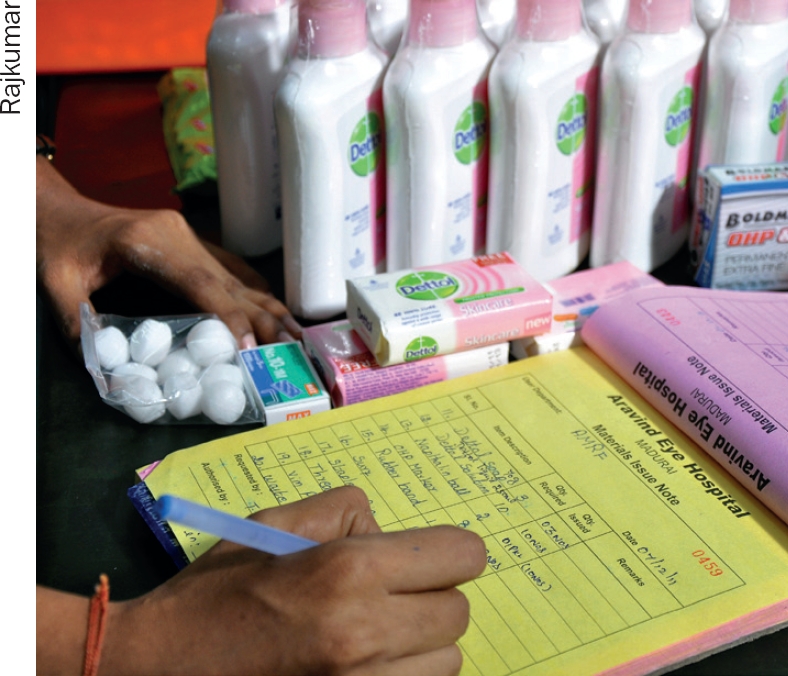
Issuing goods to user departments against a requisition form

**Worked example: re-ordering level of 10-0 sutures**

The hospital's monthly consumption of 10-0 sutures varies between 75 and 150 units a month, with an average monthly consumption of 100 units. When an order is placed, it takes two to six weeks to receive the supply with an average delivery time of 3 weeks.

**At what stock level should the hospital order 10-0 sutures?**

Since it could take as long as six weeks to get the sutures, the hospital should have adequate stocks at the time of ordering to last the six weeks it could take to receive them from the supplier. Since the hospital uses between 75 and 150 sutures a month, the consumption during the six-week period (1.5 months) could be as high as 225 (150 times 1.5). So this (225) becomes the minimum stock level, or ‘re-ordering level,’ of 10-0 sutures.

**How many 10-0 sutures should the hospital order?**

If the decision is to order a month's supplies at a time, then the order quantity would be the average monthly consumption, which is 100 sutures. Every time the stock of 10-0 sutures reaches a level of 225, an order will therefore be placed for 100 sutures. An exception would be when you know that something special will take place in the coming month, such as a big outreach event which will result in 250 more operations. In this instance, the number of sutures required to perform the additional 250 operations should be added to the routine order quantity of 100.

Similar items, such as the same medicines purchased at different times, should be kept together, with a stock card showing the quantity in stock and relevant purchase and expiry dates.

Drugs can be stored in alphabetical order or according to their use. Arrange them according to their expiry dates, with the earliest expiry dates at the front.

Depending upon the number and types of items that has to be stocked, you will need adequate shelf and floor space. Consider the following:

- Are our supplies safe and secure? Is there easy access to fire fighting equipment?
- Are the supplies protected against damage from rodents, insects, etc.?
- Does the storage area protect our supplies from damp and heat?
- Do the shelves clearly show the names of the items stored on them?
- Are we able to keep delicate items in a safe place where access is limited?
- Are we able to store chemicals for cleaning, and dangerous substances, separately, according to legal requirements? Are they clearly marked?

The storage facility should ideally be in an area which can be secured properly. In very small eye centres, a few cupboards, which can be locked, would be enough. In larger eye centres, you will need separate storage rooms. The staff managing the inventory (including the stores manager) should be based either within, or close to, the storage area.

## Inventory management

In order to manage your inventory effectively, do the following regularly:

- **Stock taking.** This ensures that the stock as per the record tallies with the physical count. Do this at least once a week. However, in a busy eye unit, it would be worth reconciling the stock every day, noting those items that have been received or issued during the day. This is also a good opportunity to note items nearing their expiry date.
- **Stock valuation.** Add up the monetary value of the stock so you know how much of your eye unit's cash is invested in stock. If you are trying to minimise costs, you can calculate the cost of consumables per patient, which is helpful to know.
- **Review of non-moving and slow-moving items.** These are any items that have not been issued for two months, or stock levels above what is needed for two months. You must determine whether these items are not required and decide what to do. Do this in consultation with the ophthalmologists and management team, perhaps when standardising your supplies. Not doing so means that items will take up valuable space and tie up funds required for other items.

Although it is vital to avoid running out of stock, we have seen that having more stock is not always the answer, since it requires more space, more investment, and more time to manage. There is also a greater chance of wastage.

It can be difficult to strike the right balance, but good record keeping, careful planning, and honest evaluation of your successes and failures will help you to work out the right stock management plan for your eye unit's needs.

**Equipment spares**

For each piece of equipment in use in your eye unit, determine which components you must replace periodically, and how often.

Take into account the time and cost involved in importing spare parts, and plan to stock adequate quantities of each item to avoid delays, should you have to replace a part.

It makes sense to purchase essential spare parts while purchasing the equipment, as the manufacturers' support, and certainly their interest, will diminish with time. It is also worth negotiating with them to provide a certain quantity of spares free of charge.

Manufacturers may try to make the spares specific to the equipment by some minor alterations or by simply renaming a generic equivalent — and charging up to four times more for it! This is particularly true of bulbs for operating microscopes and slit lamps: their generic equivalents can often be found in the automobile industry. A good knowledge of where to source generic equivalents can help you to reduce costs and establish a local source of supply.

